# Aberrant KDM5B expression promotes aggressive breast cancer through MALAT1 overexpression and downregulation of hsa-miR-448

**DOI:** 10.1186/s12885-016-2108-5

**Published:** 2016-02-25

**Authors:** Oluwaseun Adebayo Bamodu, Wen-Chien Huang, Wei-Hwa Lee, Alexander Wu, Liang Shun Wang, Michael Hsiao, Chi-Tai Yeh, Tsu-Yi Chao

**Affiliations:** Graduate Institute of Clinical Medicine, College of Medicine, Taipei Medical University, Taipei City, Taiwan; Department of Medical Research & Education, Taipei Medical University-Shuang Ho Hospital, New Taipei City, Taiwan; Department of Thoracic Surgery, Mackay Memorial Hospital, Taipei, 10449 Taiwan; Department of Pathology, Taipei Medical University-Shuang Ho Hospital, Taipei, Taiwan; Graduate Institute of Translational Medicine, Taipei Medical University, Taipei City, Taiwan; The PhD Program of Translational Medicine, Academia Sinica, Nankang, Taipei, Taiwan; Genomics Research Center, Academia Sinica, Taipei, Taiwan; Tri-Service General Hospital, Neihu District, Taipei City, Taiwan

**Keywords:** hsa-miR-448, KDM5B, MALAT1, Histone demethylase, Long non-coding RNA, microRNA, Triple negative breast cancer, Epigenetics, Anticancer target

## Abstract

**Background:**

Triple negative breast cancers (TNBC) possess cell dedifferentiation characteristics, carry out activities connate to those of cancer stem cells (CSCs) and are associated with increased metastasis, as well as, poor clinical prognosis. The regulatory mechanism of this highly malignant phenotype is still poorly characterized. Accruing evidence support the role of non-coding RNAs (ncRNAs) as potent regulators of CSC and metastatic gene expression, with their dysregulation implicated in tumorigenesis and disease progression.

**Methods:**

In this study, we investigated TNBC metastasis, metastasis-associated genes and potential inhibitory mechanisms using bioinformatics, tissue microarray analyses, immunoblotting, polymerase chain reaction, loss and gain of gene function assays and comparative analyses of data obtained.

**Results:**

Compared with other breast cancer types, the highly metastatic MDA-MB-231 cells concurrently exhibited increased expression levels of Lysine-specific demethylase 5B protein (KDM5B) and long non-coding RNA (lncRNA), MALAT1, suggesting their functional association. KDM5B-silencing in the TNBC cells correlated with the upregulation of hsa-miR-448 and led to suppression of MALAT1 expression with decreased migration, invasion and clonogenic capacity in vitro, as well as, poor survival in vivo. This projects MALAT1 as a mediator of KDM5B oncogenic potential and highlights the critical role of this microRNA, lncRNA and histone demethylase in cancer cell motility and metastatic colonization. Increased expression of KDM5B correlating with disease progression and poor clinical outcome in breast cancer was reversed by hsa-miR-448.

**Conclusions:**

Our findings demonstrate the critical role of KDM5B and its negative regulator hsa-miR-448 in TNBC metastasis and progression. Hsa-miR-448 disrupting KDM5B-MALAT1 signalling axis and associated activities in TNBC cells, projects it as a putative therapeutic factor for selective eradication of TNBC cells.

**Graphical abstract:**
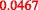
KDM5B, MALAT1 and hsa-miR-448 are active looped components of the epigenetic poculo mortis in aggressive breast cancer.

**Electronic supplementary material:**

The online version of this article (doi:10.1186/s12885-016-2108-5) contains supplementary material, which is available to authorized users.

## Background

Breast carcinoma is a huge socio-economic and clinical challenge, being the second most diagnosed cancer for both sexes combined, the most prevalent female malignancy and the leading cause of female mortality worldwide [1]. Recent data approximates the global cancer mortality at 7.6 million, and breast cancer with an annual incidence of over one million newly diagnosed cases, accounts for over 6 % of this mortality [2].

Breast cancer, like many polyetiologic human pathologies, is a product of cumulative genetic, epigenetic, somatic, and endocrine aberrations. The polyaetiologism and constitutive complexity of breast cancer presents a challenge for prevention and treatment of breast malignancies. There are five subtypes of breast cancer [3], and of these, the hormone receptor-negative basal epithelial, commonly referred to as ‘triple negative breast cancer’ (TNBC), is the most aggressive, most common among younger women of African-American and Latina ancestry and has the worst clinical prognosis [4]. TNBCs are very invasive breast carcinomas lacking estrogen (ER), progesterone (PR), and human epidermal growth factor receptors (HER2) [5] and are associated with enhanced cellular proliferation, early disease recurrence, and poor overall survival [6]. However, despite increased knowledge of the aetiology and mechanism of this breast cancer type, developing an effective anti-TNBC therapeutic strategy is still a clinical challenge.

Long non-coding RNAs (lncRNAs) are transcribed RNA molecules longer than 200 nucleotides and endogenously expressed in mammalian cells. Accumulating evidence indicate that lncRNAs, once considered to be genomic anomalies and functionless, do play significant roles in both physiologic and pathologic human conditions through their regulation of defined target mRNA expression, and their post-transcriptional epigenetic modulation [7]. Following the work of Okazaki et al., which demonstrated that many mammalian transcriptome are non-protein coding and defined lncRNAs as a significant class of these transcripts [8], it is estimated that about 11 % of the approximately 180,000 large mouse transcriptome are probably protein-coding [9], and that the number of lncRNAs far exceeds protein-coding mRNAs in the mammalian transcriptome [10]. Despite the heightened interest in lncRNA biology, their precise function in cellular processes and promoter regulation remains widely undetermined, thus, our study of the functional significance of lncRNAs, their genomic role and their epigenetic regulation.

We hypothesized that the 8.6 kb lncRNA MALAT1 is epigenetically-regulated, and that this is associated with the modulation of various oncogene expression and activities including cell proliferation, migration, invasion, and metastasis. This is consistent with documented evidence that transient MALAT1 overexpression enhanced tumor proliferation in cell lines and xenograft tumor formation in nude mice, while its attenuation resulted in reduced tumorigenicity [11, 12].

KDM5B belongs to the histone lysine demethylase family, with the ability to cause transcriptional silencing by specifically demethylating di- and tri- methylated lysine 4 of histone 3 of their target genes, and is overexpressed in several carcinomas, including gastric cancer, glioma and breast cancer [13, 14]. Observing a concomitant increase in MALAT 1 and KDM5B expression as breast cancer progresses, we investigated and validated the hypotheses that MALAT 1 interacts with KDM5B, and that the MALAT1 expression is positively regulated by that of KDM5B in the highly malignant and clinically challenging TNBC. In addition, since highlighting the problem without proffering a solution was not the intent of our work, we systematically screened for an effective therapeutic approach that not only targets KDM5B or MALAT1 expression and/or activities, but also improve clinical outcome, using a combination of small molecule inhibitors, genetic ablation or sncRNA.

## Methods

### Tissue samples

Twenty human breast tumor tissue samples collected during reduction mammoplasty and classified into various histological subtypes were obtained from archived samples of the Tissue Bank at Taipei Medical University- Shuang Ho Hospital (TMU-SHH). All of the patients gave signed, informed consent for their tissues to be used for scientific research. Recommendations of the Declaration of Helsinki for biomedical research involving human subjects were also followed. Ethical approval for the study was obtained from Joint Institutional Review Board of the Taipei Medical University (approval number: 201202007/ B201112003).

### Cell lines and culture

The panel of selected cell lines used in this study consisted of non-tumorigenic MCF-10A and non-metastatic MCF-7 breast myoepithelial cell lines, as well as six breast carcinoma cell lines, MDA-MB-231, MDA-MB-453, HS578T, T47D, AU565 and SKBr3. All cell lines were obtained from the American Type Culture Collection (ATCC, Manassas, VA, USA) and cultured according to established standard conditions using RPMI1640 supplemented by 5 μg/ml insulin (Invitrogen, Thermo Fisher Scientific Inc., Grand Island, NY, USA), 10 ng/ml EGF (Sigma), 10 % FBS (Sigma), Penicillin/Streptomycin (Sigma) in a humidified 5 % CO2 incubator. Cells were passaged at 90 % confluence and the medium changed every 48–72 h.

### KDM5B knockdown and overexpression

For KDM5B knockdown, MDA-MB-231 cells were infected with KDM5B short hairpin RNA (shRNA, clone ID -TRCN0000329952) targeting the sequence ATCGCTTGCTTCATCGATATT, GTGCCTGTTTACCGAACTAAT, or GCACCAAATTAGAGAGTCT, for clones I, II or III respectively, or vector (pLKO_TRC005), from National RNAi Core Facility, Academia Sinica, Taiwan, then shRNA expressing cells were selected with 1ug/ml puromycin. KDM5B overexpression in MCF10A cells was via transfection of the human KDM5B (NM_006618.3) cDNA sequence cloned into pCMV6-Entry vector (pCMV-KDM5B; E2384, GeneCopoeia, Inc. Rockville, MD, USA) using LipofectAMINE PLUS reagent (Life Technologies, Thermo Fisher Scientific Inc., NY, USA). MCF10A cells were seeded and cultured in 35 mm diameter dishes until 80 % confluence. On the day of transfection, 1 mg of DNA diluted in 100 μl of serum-free medium, and 6 μl of LipofectAMINE PLUS regents were then added. The DNA-PLUS mix was incubated for 20 min at room temperature, and then 4 μl of LipofectAMINE reagent was added and incubated for an additional 20 min. After incubation, the cells were washed with serum-free medium twice and 800 μl of serum-free transfection medium. The DNA-PLUS–LipofectAMINE reagent mix was then added to the cells and incubated at 37 °C in 5 % humidified CO_2_ incubator for 3 h. After 3 h, recovery medium with 10 % FBS was added till final volume of 2 ml and incubated. After incubating overnight, the recovery medium was suctioned and fresh DMEM medium containing serum and antibiotics added.

### RNA extraction, RT-PCR and real time PCR

Total RNA was isolated using TRI Reagent (Sigma) according to manufacturer’s protocol. RNeasy Mini Kit was used for RNA purity optimization. Total RNA concentration was determined using NanoDrop ND1000 spectrophotometer (Nyxor Biotech, Paris). 1 μg of total RNA was transcribed reversely with 2 μg of random hexamers (Amersham, Taipei, Taiwan) and Superscript III reverse transcriptase (Invitrogen, Thermo Fisher Scientific Inc., Grand Island, NY, USA) according to manufacturer’s instructions. DEPC-treated water was used to dilute cDNA 100 folds and stored at −20 °C. Real-time PCR was done using SYBR Green PCR Master Mix using inbuilt System Software (Applied Biosystems, Life Technologies, Grand Island, NY, USA), 200nM forward and reverse primers, and cDNA equivalent of 0.5ug RNA. The triplicate PCR reaction conditions were as follows: 25 °C–5 min, 42 °C–60 min, 70 °C–5 min; total 45 cycles of 70 °C–10 min. 20ul PCR product was loaded to 1.5 % SYBR Green agarose gel for electrophoresis and checked under UV light. Gene expression was normalised to GAPDH and altered expression measured relative to the control (MDA-MB-231 Vector; shKDM5B-vector infected MDA-MB-231 cells).

### Western blot analysis

Total cell lysates were prepared and analyzed by western blot assay. Primary antibodies used included KDM5B polyclonal antibody (H00010765-A01; Abnova, Neihu District, Taipei City, Taiwan), Oct 4 Rabbit mAb (C30A3; Cell Signaling Technology, Inc., Beverly, MA, USA), Survivin (FL-142: sc-10811; Santa Cruz Biotechnology, Inc., Dallas, Texas, USA) and GAPDH. Secondary antibodies were Alexa Fluor 680-conjugated affinity-purified anti-mouse or anti-rabbit IgG (Invitrogen, Thermo Fisher Scientific Inc., Grand Island, NY, USA) detected using the UVP Imaging.

### Colony formation assay

2 × 10^4^ cells were seeded into a 6-well cell culture plate and incubated for 2 weeks at 37 °C after treatment. Then, cells were washed twice with PBS, fixed with cold methanol, stained with 0.005 % crystal violet, washed and air dried. Colonies were then counted. In each well, the total colonies with a diameter ≥ 100 μm were counted over 5 randomly selected fields in triplicate assays.

### Matrigel invasion assay

Using the 24-well plate Transwell system, 3 × 10^4^ cells were seeded into the upper chamber of the insert (BD Bioscience, 8 μm pore size) containing medium without serum, and medium containing 10 % FBS in the lower chamber served as chemoattractant. After 24 h of incubation, medium was discarded, cells on filter membrane were fixed with 3.7 % formaldehyde for 1 h and stained with crystal violet staining solution, and cells on the upper side of the insert were removed with a cotton swab. The migrated cells were visualized and migratory capacity was evaluated as the total number of cells on the lower surface of the membrane, as determined by microscopy.

### MiRNA profiling and secondary structure prediction

We used TargetScan [15], PicTar [16] and miRANDA [17] for miR profiling and target sorting. The M-FOLD program v 2.3, [18] was employed to predict the secondary structure of hsa-miR-448. The prediction was done as earlier described by Bellucci M and colleagues [19].

### Access and probe of online cancer data set

Publicly available and freely accessible online cancer data repositories used in this study include TCGA, Oncomine, GEO and CCLE. The Cancer Genome Atlas (TCGA) dataset used was the breast invasive carcinoma (BRCA)-IlluminaHiSeq RNAseq, *N* = 1182 [20]. We downloaded and analyzed the TGCA dataset using the UCSC Cancer Browser [21] and via the Oncomine interface [22]. We also used dataset GSE3494, platforms GPL 97 from the Gene Expression Omnibus (GEO) [23] consisting of freshly frozen breast tumors from a cohort of 315 women which represents 65 % of all breast cancers resected in Uppsala County, Sweden, from 1/1/1987 to 31/12/1989,with their estrogen receptor (ER) status determined using biochemical assay.

### Immunohistochemical staining and statistical analyses

A total of 270 patients diagnosed with breast carcinoma between January 1, 2005 and December 31, 2010 in Mackay Memorial Hospital (Taipei City, Taiwan) were enrolled for the study. All of the patients gave signed, informed consent for their tissues to be used for scientific research. Recommendations of the Declaration of Helsinki for biomedical research involving human subjects were also followed. Ethical approval for the study was obtained from Joint Institutional Review Board of the Mackay Memorial Hospital (approval number: 11MMHIS154). Patients’ clinical records were reviewed to determine tumor stage at the time of diagnosis and outcome. H&E–stained sections of the mammoplasty specimens were reviewed to select representative areas of the tumor to carry out KDM5B immunohistochemical detection. The working dilution was 1:200. KDM5B immunohistochemistry was carried out using tissue microarray (TMA) on an automated system for immunostaining (Dako Autostainer), with antigen retrieval at high pH. We graded the intensity of the membrane and cytoplasmic staining as absent, weak, moderate, or intense, after stained sections were counterstained with hematoxylin. However, for subsequent statistical analysis we reclassified the cases as high (moderate or intense) or low (null or weak staining similar to control areas of normal breast tissue). In all cases, sections from normal breast tissue bordering the tumor site were used as negative controls. We carried out survival analysis using the Cox univariate and multivariate analyses of proportional hazards model for KDM5B status and selected clinicopathological predictors of outcome. The multivariate model was produced by assessing KDM5B status with other baseline covariates of clinical relevance, such as tumor size, body weight, lymph node metastasis, and hormone receptor status. Log-rank test was used to evaluate significant survival probability differences, while 95 % confidence interval (CI) and hazard ratio (HR) were derived from the regression coefficients. Data were expressed as mean ± standard error of mean, and compared using one way ANOVA and Student’s t-test. *p* < 0.05 was considered statistically significant.

## Results

### Upregulation of KDM5B expression in human breast cancer tissues and cell lines

**Fig. 1:**
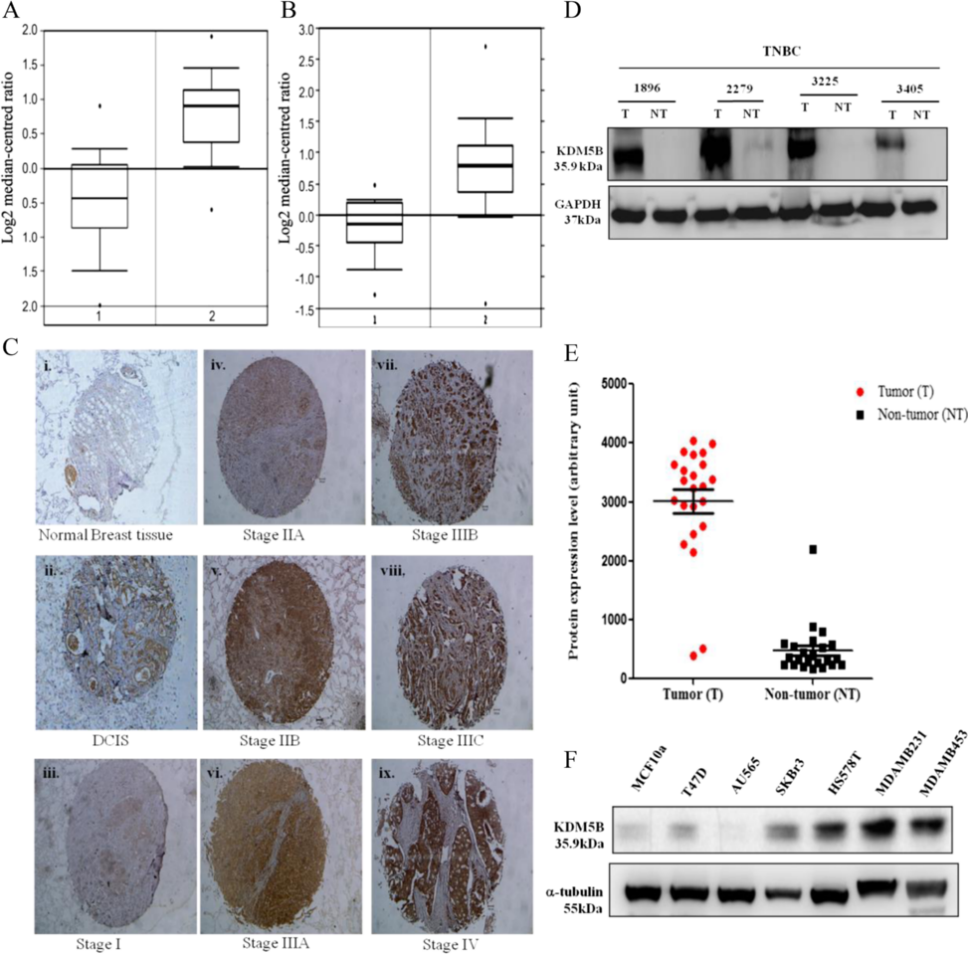
KDM5B expression is upregulated in human breast cancer tissues and cell lines. KDM5B is overexpressed in triple negative TGCA (**a**) invasive lobular breast cancer, and (**b**) invasive ductal breast cancer tissues, compared to their counterpart normal breast tissues. 1, normal breast tissue; 2, invasive breast carcinoma (**c**) Representative photomicrograph of immunohistochemical staining of KDM5B in human breast cancer tissues showed that KDM5B expression occurs preferentially in TNBC late tumor stage samples. Photographs were taken at 200× magnification. Western blot showing KDM5B expression in (**d**, **e**) paired tumor: non-tumor human breast samples. **f** Western blot analysis of KDM5B expression in 7 different breast cancer cell lines. KDM5B is strongly expressed in TNBC cell lines, MDA-MB-231, MDA-MB-453 and HS-578 T. GAPDH and α-tubulin were used as loading control. Experiments were done in triplicate and quantified by ImageJ densitometry*.* KDM5B, lysine specific demethylase 5B protein; T, tumor; NT, non-tumor; DCIS, ductal carcinoma in situ; 231, MDA-MB-231; 453, MDA-MB-453

To understand the biofunctional significance of KDM5B in human breast cancer, using bioinformatics approach, we accessed and probed the TCGA invasive lobular and ductal breast carcinoma dataset consisting of 593 samples via the Oncomine interface. Analysing the expression of KDM5B in this dataset, we observed that KDM5B expression in the invasive lobular breast cancer samples was upregulated by about 2.44-fold (*p* < 0.001, Fig. 1a) compared to the normal breast tissue, while in the invasive ductal breast cancer set, KDM5B was overexpressed 1.95-folds higher than the normal breast tissue group (*p* < 0.001, Fig. 1b). Using immunohistochemical staining, we confirmed the endogenous expression of KDM5B in breast cancer samples and correlative analysis showed that KDM5B expression level positively correlated with tumor histological grade (*p* < 0.001), with highest expression in grade IV (Fig. 1c). Furthermore, our comparative analysis of the expression levels of KDM5B in 23 paired breast carcinoma and adjacent non-neoplastic tissues from TMU-SHH breast cancer patients’ cohort using western blot showed that the expression levels of KDM5B were elevated in 21 of 23 cases of breast cancer specimens compared with those of adjacent non-neoplastic tissues (*p* < 0.001) (Fig. 1d & e). In addition, we assessed the expression levels of KDM5B in breast cancer cell lines. Result indicated elevated expression level of KDM5B in the TNBC cell lines compared to their counterpart non-TNBC cell lines. The highly metastatic MDA-MB-231 and MDA-MB-453 cells exhibited the strongest expression of KDM5B (Fig. 1f). These results indicate that increased KDM5B expression is characteristic of highly malignant human breast cancer cells and tissues, as well as suggest its involvement in breast carcinoma aggressive phenotype and progression.

### High KDM5B expression is significantly associated with clinical outcome in breast carcinoma, in vivo

**Fig. 2:**
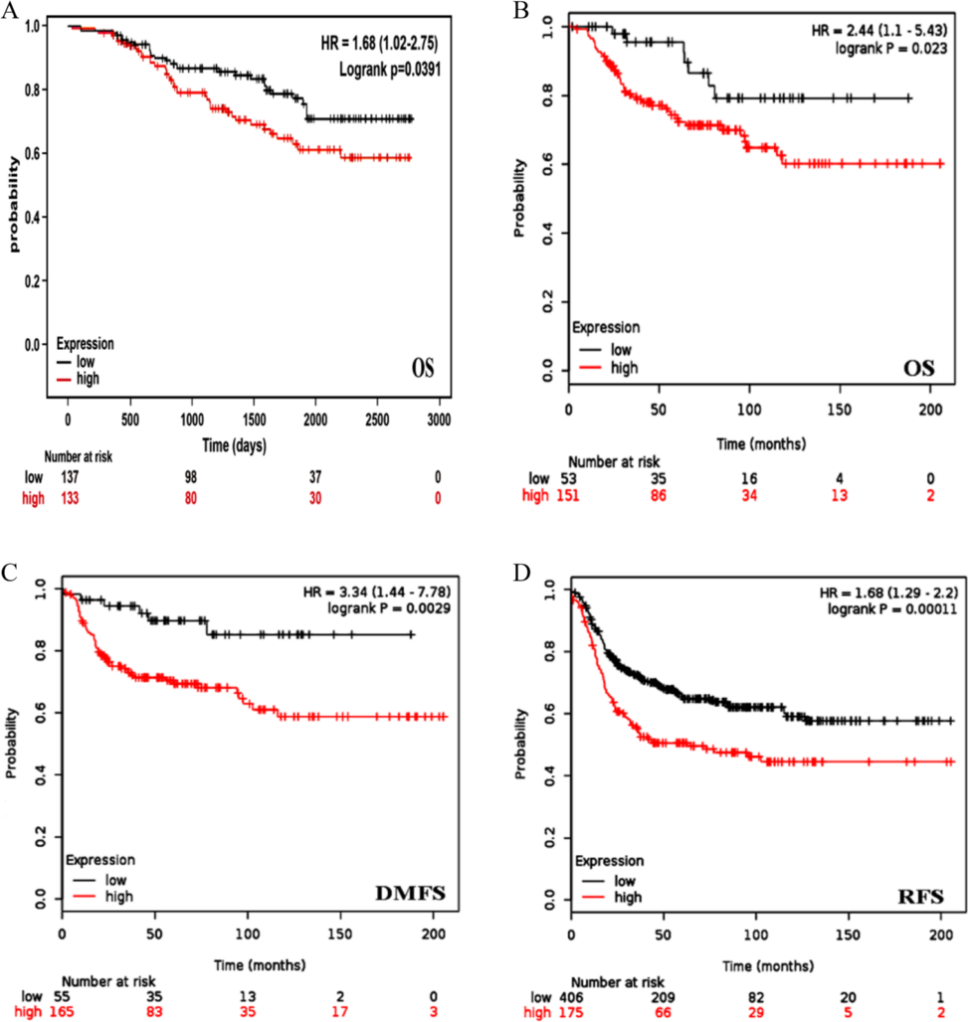
Correlation of KDM5B expression with patients’ survival. Kaplan-Meier curves with univariate analyses (log-rank) of KDM5B expression in triple negative breast cancer patients stratified as high and low KDM5B, where high KDM5B was defined as >80 % average nuclear expression, while low KDM5B <80 % nuclear expression (*P < 0.05). **a** OS according to nuclear KDM5B immunohistochemical expression in TNBC tissues from the Mackay Memorial Hospital cohort of 270 patients. **b** OS according to nuclear KDM5B immunohistochemical expression in TNBC tissues from the GEO breast cancer cohort. **c** DMFS according to nuclear KDM5B immunohistochemical expression in TNBC tissues from the GEO breast cancer cohort. **d** RFS according to nuclear KDM5B immunohistochemical expression in TNBC tissues from the GEO breast cancer cohort. OS, overall survival; DMFS, distance metastasis-free survival; RFS, relapse-free survival

To evaluate the prognostic relevance of the KDM5B expression in the cohorts, cases were stratified as high and low. Our initial discovery cohort consisting of only 23 subjects, was considered small and lacked proper documented follow-up, therefore, the relationship between KDM5B expression and tumor progression using the initial cohort data was subjected to further exploration in a larger, independent validation cohort of 270 breast cancer patients TMA with longer follow-up, which included overall disease-specific survival. In our validation cohort, KDM5B was strongly expressed in 49.2 % (*n* = 270) of the breast cancer samples (Fig. 2a). Both univariate and multivariate analyses using the Cox proportional hazards model showed that KDM5B expression, lymph node metastasis, and tumor size were all significant predictors of breast cancer biochemical and clinical outcome (Table 1). *χ*^2^ correlative analysis of the KDM5B expression and clinicopathological parameters revealed significant associations between high KDM5B expression and larger tumor size (*p* = 0.005, Table 2). Patients with higher KDM5B expression had significantly poorer prognosis compared to those with low expression, as assessed by overall survival (multivariate: HR 1.68, 95 % confidence interval (CI) 1.02–2.75, *p* = 0.04; univariate: HR 1.63, 95 % confidence interval (CI) 1.01–2.65, *p* = 0.047, Table 1). Adjunct to our data, we further analysed triple negative breast cancer prognosis data obtained from the gene expression omnibus, GEO – Affymetrix HGU133A and HGU133 + 2, consisting of 1809 patients [14]. Consistent with our findings, in multivariate analysis, high KDM5B expression profile was a significant predictor of poor overall survival, OS (HR 2.44, 95 % CI 1.1–5.43, *p* = 0.023), distance metastasis-free survival, DMFS (HR 3.34, 95 % CI 1.44–7.78, *p* = 0.0029) and relapse-free survival, RFS (HR 1.68, 95 % CI 1.29–2.2, *p* = 0.00011, Fig. 2b–d), projecting KDM5B overexpression as an independent predictor of poor clinical outcome.

**Table 1:** Univariate and Multivariate analyses using Cox proportion hazards model of overall survival of all hormone negative breast cancers (*n* = 270)

	Univariate analysis				Multivariate analysis			
Parameters	HR	95 % CI		*P* value	HR	95 % CI		*P* value
KDM5B expression High vs Low	1.635	1.007	2.653		1.678	1.025	2.749	
Tumor size	1.009	0.999	1.02	0.0796	1.011	0.999	1.023	0.0761
Body weight	1.001	0.975	1.028	0.9378	0.998	0.972	1.025	0.9059
Height	1.021	0.993	1.05	0.136	1.025	0.995	1.055	0.1005
BMI	1.001	0.873	1.147	0.99	1.054	0.915	1.215	0.4625
Lymph nodes metastasis	1.016	1.001	1.031		1.027	1.006	1.048	
ER status	1.447	0.889	2.354	0.1371	1.395	0.839	2.318	0.1994
HER2 status	0.907	0.548	1.499	0.702	0.835	0.493	1.414	0.5014

Red indicates *p* value < 0.05

*HR* hazard ratio, *CI* confidence index, *BMI* body-mass index, *ER* estrogen receptor, *HER2* human epidermal growth factor receptor 2

**Table 2:** Correlation between KDM5B expression and clinicopathological parameters

Parameter	Low KDM5B expression	High KDM5B expression	*χ* ^2^ value	*p*-value
Tumor size				
≤3	85 (62.04 %)	60(45.11 %)	7.78	
>3	52(37.96 %)	73(54.89 %)		
Lymph nodes				
≤18	65(47.45 %)	65(48.87 %)	0.06	0.814
>18	72(52.55 %)	68(51.14 %)		

Red indicates p value < 0.05

### KDM5B enhances the proliferation and maintenance of aggressive breast cancer cells malignant phenotype

**Fig. 3:**
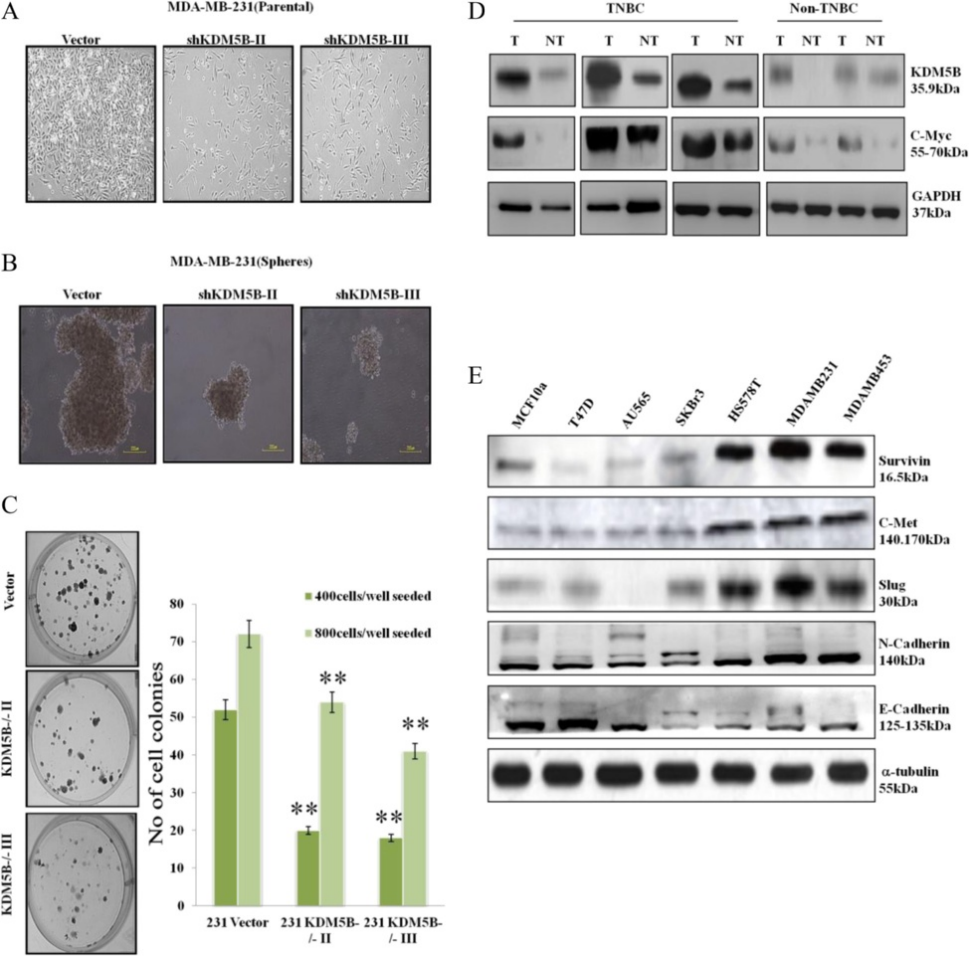
KDM5B enhances the proliferation and maintenance of TNBC cells malignant phenotype. **a** Proliferation assay showing that KDM5B knockdown inhibited cell proliferation, (**b**) silencing KDM5B suppressed the ability of the shKDM5B infected MDA-MB-231 cells to form mammospheres, and (**c**) colony formation assay showed that vector-infected cells possessed greater clonogenicity than their counterpart shKDM5B infected MDA-MB-231 cells. The experiments were carried out in triplicate. Scale bar: 200 μm. **d** Western blot showed that in human breast tissue samples KDM5B expression is higher in the TNBC tissues, compared to their non-TNBC counterparts and (**e**) the expression profile of KDM5B in different breast cancer cell lines, is similar to that of Survivin, c-Met, slug, c-Myc and N-cadherin, and converse to that in E-cadherin. GAPDH and a-tubulin were used as loading control. KDM5B, lysine specific demethylase 5B protein; 231, MDA-MB-231; 453, MDA-MB-453; **P < 0.01

To investigate the effect of KDM5B on the proliferation and malignant phenotype of breast carcinoma cells, we performed cell proliferation, mammosphere formation and colony formation assays. We confirmed previous reports that the proliferation of breast cancer cells was inhibited by the conditions in which KDM5B expression and/or activity was attenuated on the premise that increased invasiveness of the triple negative breast cancer cells was augmented by proliferation of these cancer cells. There was increased proliferation (Fig. 3a), mammosphere generation (Fig. 3b) and colony formation (Fig. 3c) in the KDM5B-positive vector breast cancer cells, while cells with attenuated KDM5B expression exhibited reduced proliferation, mammosphere formation and clonogenicity ability. To determine if KDM5B was sufficient for the activation of the increased TNBC oncoagression observed, we further evaluated the expression of KDM5B, c-Myc (a metastatic factor and mesenchymal marker) and other EMT markers in breast cancer tissues and cell lines. Our data revealed a similar expression pattern for KDM5B and c-Myc in both clinical samples (Fig. 3d). Positive correlation was also noted for the expressions of KDM5B and the survival factor, Survivin, as well as with EMT markers, c-Met, Slug and N-Cadherin, while as anticipated E-Cadherin exhibited a reverse pattern of expression compared with KDM5B (Fig. 3e). Thus, we inferred that the difference in the malignant invasive potential of the breast cancer cells was due to their differential KDM5B expression.

### Silencing of KDM5B markedly reduced migration and invasive potential of TNBC cells

**Fig. 4:**
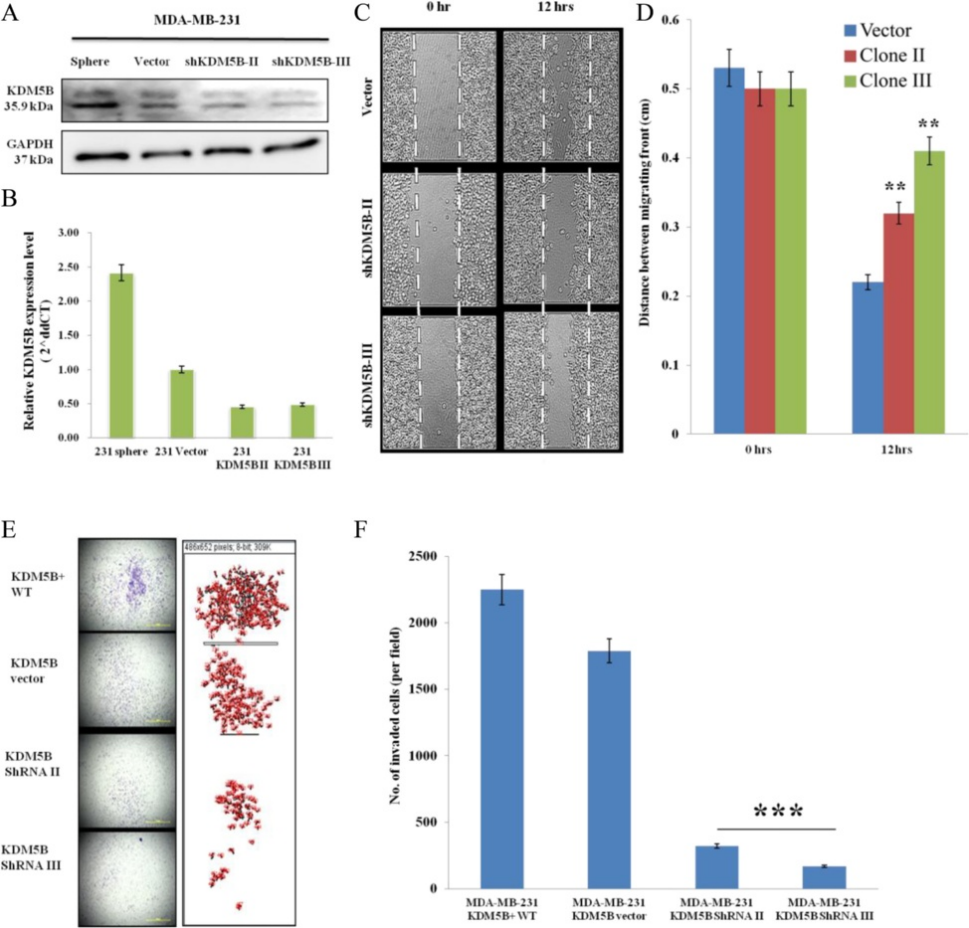
Silencing of KDM5B markedly reduces the migration and invasive potential of TNBC cells. **a**, **b** KDM5B expression in MDA-MB-231 mammosphere, cells stably expressing KDM5B vector or shKDM5B assessed by western blot analysis and RT-PCR. GAPDH serves as loading controls. **c**, **d** MDA-MB-231 control vector or KDM5B-depleted cells were subjected to a wound healing in vitro *migration* assay as described under Materials and methods. Representative photomicrographs at indicated time points from three independent experiments, each performed in triplicate wells, are shown. Magnification: × 20. The cells were allowed to migrate after wounding for 12 h. The extent of wound recovery was determined by measuring the distance between migrating cellular fronts at 5 randomly selected points and finding the average. Migration was significantly inhibited in shKDM5B-expressing MDA-MB-231 cells as compared with that in control wild-type cells. Column: Mean of three experiments; bar: standard error, *p*-value was determined by student’s *t*-test (*P < 0.05; ***P < 0.001). **e**, **f** KDM5B ablation in MDA-MB-231 cells significantly attenuated invasion of shKDM5B MDA-MB-231 cells as compared to control wild-type and vector cells. Column: Mean of three experiments. Bar: standard error *P*-value was determined by Student’s *t*-test (***P < 0.001). KDM5B, lysine specific demethylase 5B protein; shRNA, short hairpin RNA

KDM5B-expressing TNBC cells showed enhanced cell invasion in the transwell matrigel invasion system. Using the short hairpin RNA (shRNA) gene-silencing approach, we determined the knockdown efficiency of KDM5B at both protein (Fig. 4a) and messenger (Fig. 4b) levels. We evaluated the effect of KDM5B expression on tumor cell motility with or without KDM5B knockdown. There was a significant lag in migration (Fig. 4c, d) and invasiveness (Fig. 4e, f) in the KDM5B-silenced MDA-MB-231 cells, compared to vector control. These data indicate that KDM5B enhances the invasion and migration of TNBC cells, while its attenuation genetically or via small molecular inhibitors has a converse effect.

### Forced KDM5B expression induced tumorigenicity, enhanced migration, and acquisition of CSC-like phenotype in non-tumorigenic MCF-10A breast cancer cell line

**Fig. 5:**
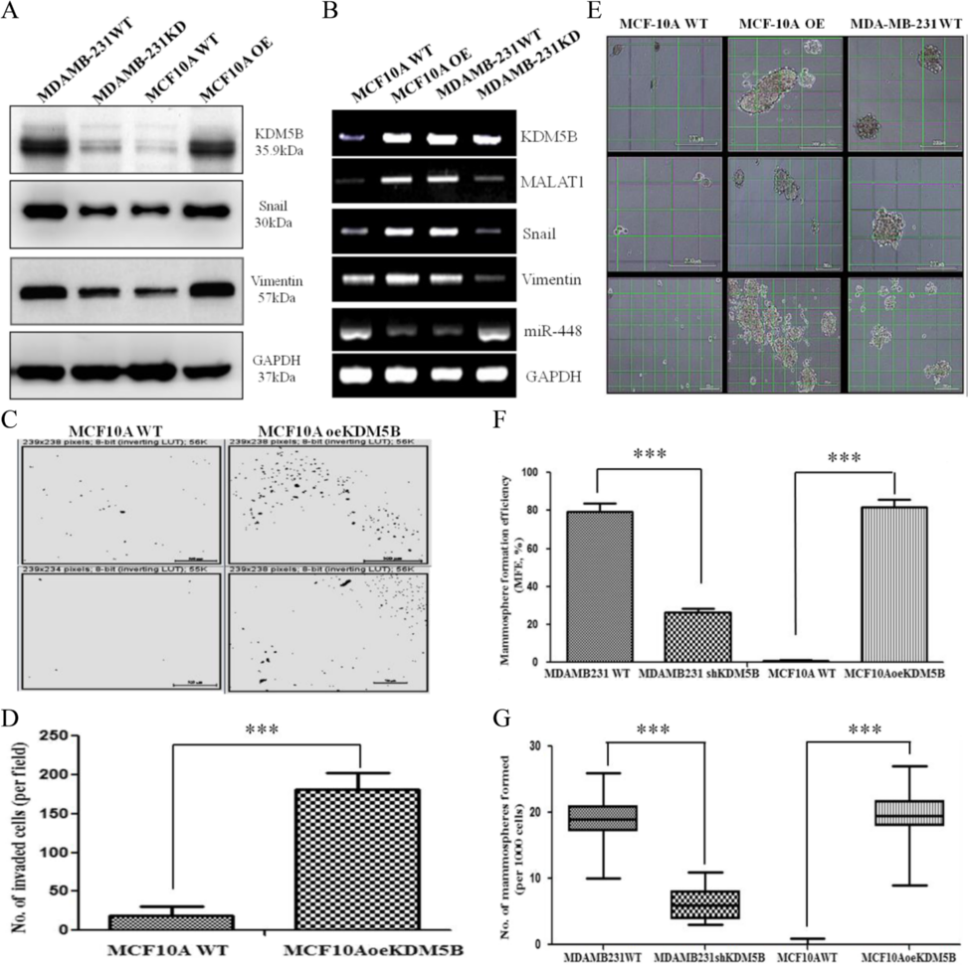
Forced KDM5B expression induced tumorigenicity, enhanced migration, and acquisition of CSC-like phenotype in non-tumorigenic MCF-10A breast cancer cell line. **a** Western blot showing cytoplasmic and nuclear expression profile of KDM5B compared with that of snail and vimentin in MDA-MB-231 WT, MDA-MB-231 KD, MCF10A WT and MCF10A OE. **b** A representative RT-PCR result showing transcript expression of KDM5B, MALAT1, snail, vimentin, and miR-448. GAPDH served as loading or internal control. **c** Mammosphere formation assay showing the effect of induced KDM5B expression in MCF10A cells. MCF10A WT and MDA-MB-231 WT cells were used as negative and positive control, respectively. **d**, **e** Quantitative box-plots depicting mammosphere formation ability and efficiency in MDA-MB-231WT, MDA-MB-231shKDM5B, MCF10AWT and MCF10AoeKDM5B. **f**, **g** Representative Photomicrogram and bar chart of invasion assays showing the effect of KDM5B overexpression in MCF10A cells. ***P < 0.001

In analogous experiments, we investigated the effect of induced KDM5B expression on the oncogenic potential of non-tumorigenic MCF-10A myoepithelial cells, using the western blot, transwell matrigel invasion and mammosphere formation assays. We observed that induced expression of KDM5B yielded an upregulation of snail and vimentin protein expression (Fig. 5a), as well as approximately 1.58- and 1.45- fold increase in snail and vimentin transcript expression in the MCF-10AoeKDM5B cells relative to MCF-10A WT. In addition, MALAT1 transcript expression and/or activity were upregulated in the MCF 10AoeKDM5B cells 2.18-folds when compared with their un-induced MCF-10A WT counterparts (Fig. 5b, Additional file 1: Table S1 and Additional file 2: Figure S1). We noted that induction of KDM5B expression in the MCF-10A cells (MCF-10AoeKDM5B) enhanced their migratory ability by approximately 10-fold in comparison to their wild type counterpart, MCF-10A WT (Fig. 5c, d). Of note is our finding that forced KDM5B expression in MCF-10A OE cells induced the acquisition of cancer stem cell-like phenotype in the KDM5B-deficient, non-tumorigenic MCF-10A cells, as evidenced by their markedly increased ability to form multiple large-sized mammospheres, analogous to those formed by the highly malignant TNBC cell, MDA-MB-231, which served as our positive control (Fig. 5e). The mammosphere formation efficiency (MFE) of the MCF10A WT was significantly augmented by induced expression of KDM5B from 10 ± 0.004 % to 82 ± 0.04 %, while as anticipated the MFE of MDA-MB-231 WT cells in the mammosphere culture was markedly suppressed by shKDM5B from 79 ± 0.04 % to 26 ± 0.02 % (*p* < 0.001) (Fig. 5f). In addition, the size of MDA-MB-231-derived mammospheres decreased by shKDM5B, as evidenced by significantly lesser number of proper mammospheres (>200 μm) from 19.0 ± 4.13 to 6.39 ± 2.37 (*p* < 0.001), while on the other hand, that of the MCF10AoeKDM5B increased to 19.63 ± 0.04 (*p* < 0.001), compared to the 0.21 ± 0.4 in its wild type counterpart (Fig. 5g). This is consistent with our earlier findings that suggested that KDM5B plays a critical role in the formation and maintenance of breast cancer stem cells (BCSCs). Collectively, these data indicate that KDM5B expression is sufficient for induction of pluripotency in breast, critical for self-renewal of breast tumor cells, effectively activates metastatic factors, initiates EMT and invariably is crucial for the BCSCs-mediated invasiveness of hitherto non-tumorigenic MCF10A cells.

### KDM5B induces the expression of MALAT1 and its effector metastasis- associated genes in triple negative breast carcinoma cells

**Fig. 6:**
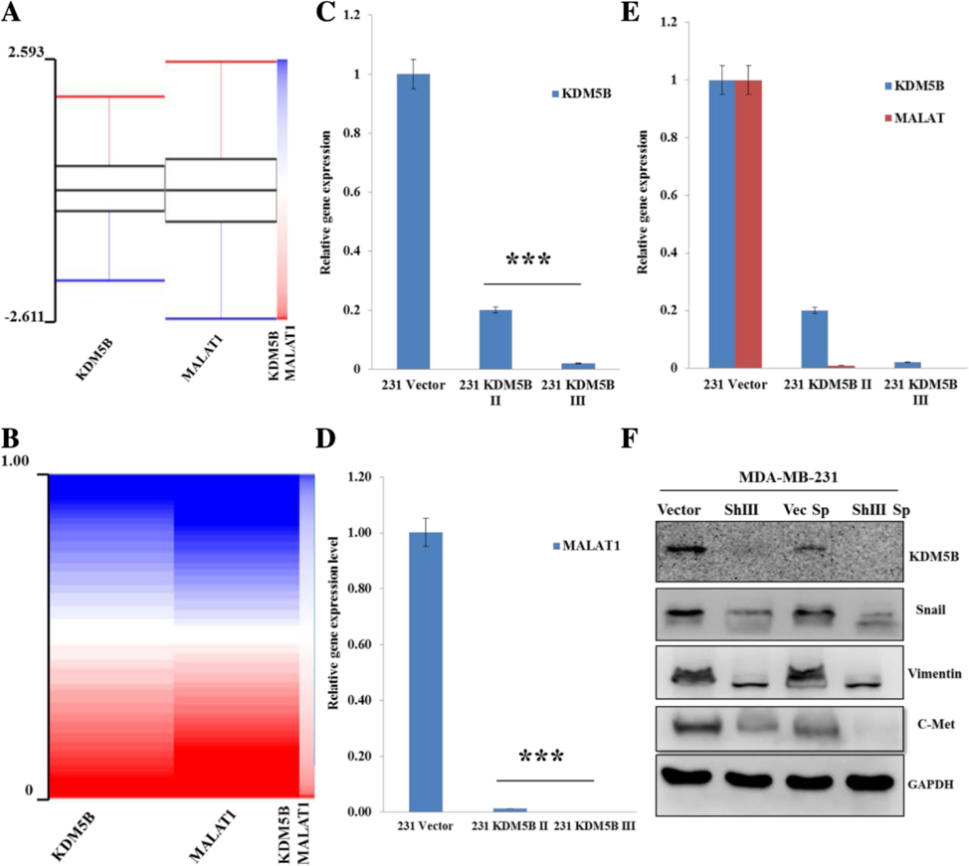
KDM5B induces the expression of MALAT1 and its effector metastasis-associated genes in TNBC cells. **a**, **b** TCGA illuminaHiSeq breast invasive carcinoma dataset analysis show similar KDM5B and MALAT1 gene expression levels and proportion in 1182 clinical samples probed. **c**–**e** The effect of KDM5B knockdown on the expression of KDM5B or MALAT1 transcripts using quantitative RT-PCR. **f** Western blot of control, shKDM5B transfected, vector-generated mammospheres and shKDM5B-III transfected spheroid cells. Cellular lysates containing 20 μg of total protein were subjected to western blotting using the indicated antibodies. The data are representative of triplicate experiments. ***p < 0.001; 231 KDM5B II/III, shKDM5B infected MDA-MB-231 clone II/III cells; ShIII, shKDM5B infected MDA-MB-231 clone III cells; ShIII Sp, mammospheres generated from shKDM5B infected MDA-MB-231 clone III cells; Vec Sp, mammospheres generated from vector infected MDA-MB-231 cells

Our data suggest a critical role for MALAT1 in the KDM5B-mediated malignant phenotype in TNBC cells. In parallel experiments, we sought to verify our observation that KDM5B acts as an epigenetic regulator of MALAT1, and MALAT1 in turn, modulates downstream snail and vimentin. Employing bioinformatics approach, we evaluated the correlative associatedness of KDM5B and MALAT expression in malignant breast cancer. We probed the TCGA illuminaHiSeq breast invasive carcinoma dataset consisting of 1182 human breast tumor cases, and demonstrated notable similarity in gene expression levels and proportion in the probed samples (Fig. 6a & b), while our qRT-PCR demonstrated that downregulation of KDM5B expression via shRNA induced a significant concomitant suppression of MALAT 1 expression (Fig. 6c–e). Using same database, we performed a comparative evaluation of the expression profile of KDM5B, MALAT1 and selected metastasis, stemness, survival and drug resistance genes in TNBC and non-TNBC. This showed a preferential expression of KDM5B and its associated effector genes or substrates, in TNBC compared to their non-TNBC counterparts (Additional file 3: Figure S2). Furthermore, our western blot results corroborated the bioinformatics and qRT-PCR findings, showing that KDM5B signalling induced MALAT1 activity and consequently induced the expression of the metastasis-associated genes, snail and vimentin in the vector control (Vec) and spheroid (Vec Sp) cells, compared to their KDM5B-silenced counterparts (shIII and shIII Sp) (Fig. 6f). These findings are consistent with previous results where induced expression of KDM5B in MCF10A cells enhanced the expression of snail and vimentin (Fig. 5b), and thus, suggest that KDM5B promotes TNBC malignant phenotype by modulating MALAT1 activity.

### KDM5B interacts with MALAT1 to modulate its expression in triple negative breast carcinoma cells and consequently facilitate invasion and associated metastatic activities

**Fig. 7:**
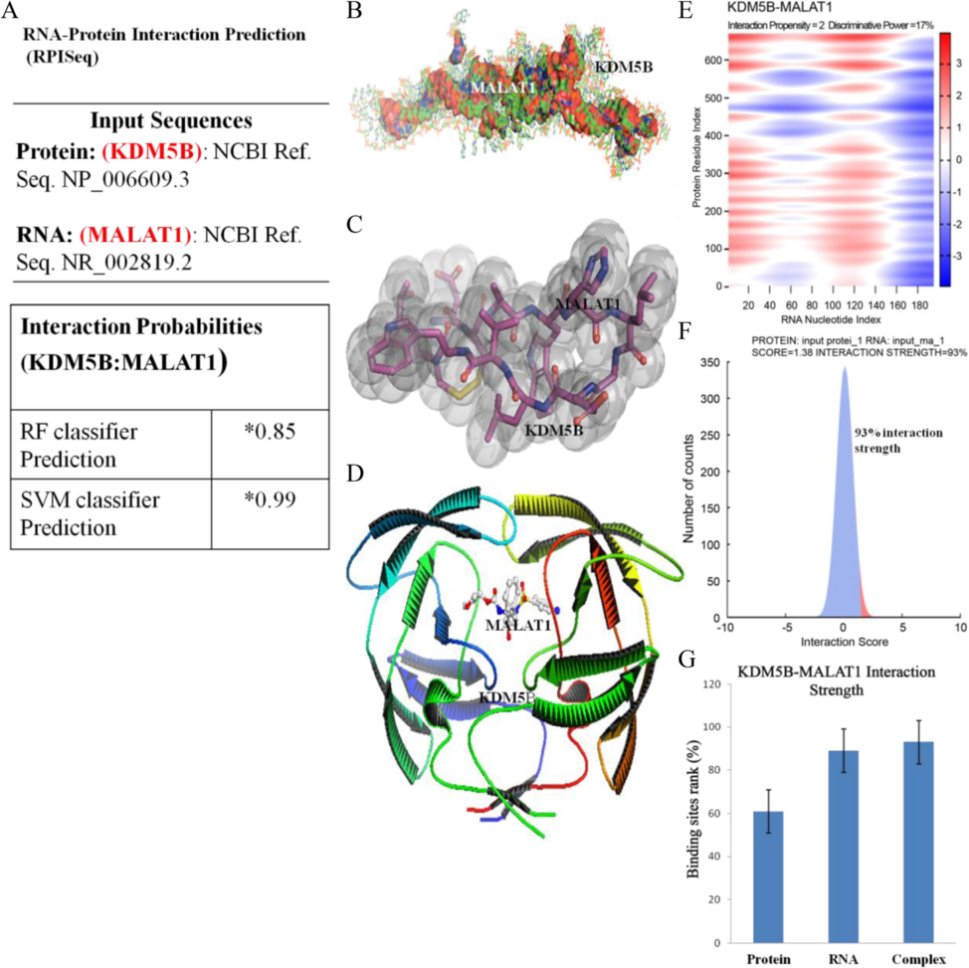
KDM5B interacts directly with MALAT1 to modulate its expression in triple negative breast carcinoma cells. **a** Sequence-based prediction of MALAT1-KDM5B interaction, with random forest, RF classifier score of 0.85 and support vehicle machine, SVM classifier score of 0.99. * Interaction probabilities generated by RPISeq range from 0 to 1. In performance evaluation experiments, predictions with probabilities > 0.5 were considered “positive,” i.e., indicating that the corresponding RNA and protein are likely to interact. **b** PyMOL view of long non-coding RNA, MALAT1 in complex with KDM5B. **c** Post-SCULPTing rendering of the KDM5B-MALAT1 complex. **d** Mol script/R3D Input representation of the complex, show KDM5B wrapped around MALAT1. **e** Heat map of KDM5B-MALAT1 interaction using the catRAPID graphic. Interaction propensity is measured in *procedure defined unit, p.d.u*. **f**, **g** Interaction strength based on interaction propensity rank of binding regions in the positive set (10201 interacting pairs) using catRAPID strength; *Error bars, S.E*

To better understand the functional association between KDM5B and MALAT1, as well as the underlying mechanism by which the later mediates the activity of the former, we assessed the propensity for KDM5B-MALAT1 interaction (Fig. 7a), the strong interactability or complex formability of KDM5B with MALAT1 using the sequence-based RNA-Protein interaction prediction (Fig. 7b–d), and the strength of such interaction (Fig. 7e–g), patterned after recently published bioinformatics approach [24–26]. KDM5B protein sequence used was the NCBI Ref. Seq. NP_006609.3 while the sequence of MALAT1 used was NCBI Ref. Seq. NR_002819.2. We showed that based on the Random Forest (RF) and Support Vector Machine (SVM) classifier prediction, the probability of interaction between MALAT1 and KDM5B ranged between 85 and 99 % (Fig. 7a).

Further, to appreciate the architectural viability of KDM5B-MALAT1 complex formation, we carried out ligand docking coupled with limited binding site analysis using the educational-use-only version of GUI PyMOL software [27]. Figure 7b–d show alternative 3D molecular visualization of the KDM5B-MALAT1 complex. While Fig. 7c is post-SCULPTing, an interactive process that allowed us to manipulate the molecular structures of KDM5B and MALAT1 while computing associated molecular mechanics in real time, Fig. 7d is a Mol script/Raster3D input, using a rapid Z-buffer algorithm to generate high quality pixel image of the KDM5B-MALAT1 complex, with characteristic shadowing and non-shadowing light source, transparency, specular highlighting, as well as Phong-shaded molecular surfaces. We then utilized the catRAPID graphic RNA-Protein interaction validation tool [28] for validation of our data. There was significant interaction activity between KDM5B and MALAT1 based on the generated heat map (Fig. 7e), and KDM5B-MALAT1 interaction strength of 93 % (Fig. 7f & g) estimated from the interaction propensity rank of binding regions in the positive set (10201 interacting pairs) using the catRAPID strength tool.

### KDM5B is a functional target of hsa-miR-448, thus, aberrant downregulation of the later in KDM5B-overexpressing triple negative breast carcinoma tissues and cell lines

**Fig. 8:**
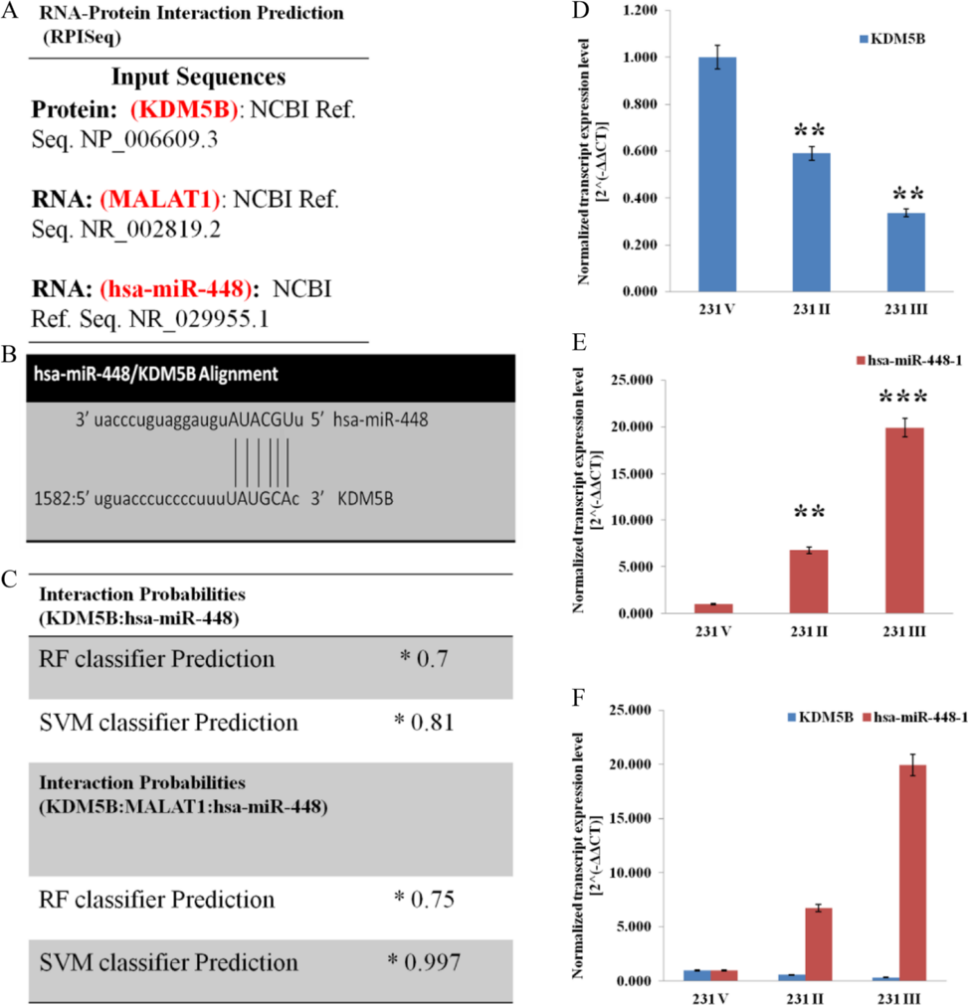
KDM5B is a functional target of hsa-miR-448 in TNBC cells. **a** KDM5B, MALAT1 and hsa-miR-448 protein or nucleotide input sequence obtained from the National Center for Biotechnology Information (NCBI) database, http://www.ncbi.nlm.nih.gov/. **b** Predicted KDM5B 3′-UTR binding site for hsa-miR-448. The hsa-miR-448 seed region alignment with KDM5B 3′-UTR is shown. **c** Upper panel: Sequence-based prediction of KDM5B-hsa-miR-448 interaction, with random forest, RF classifier score of 0.7 and support vehicle machine, SVM classifier score of 0.81. Lower panel: Sequence-based predicted interaction of KDM5B-MALAT1-hsa-miR-448, with RF classifier score of 0.75 and SVM classifier score of 0.997. * Interaction probabilities generated by RPISeq range from 0 to 1. In performance evaluation experiments, predictions with probabilities > 0.5 were considered “positive,” i.e., indicating that the corresponding RNA and protein are likely to interact. **d** The effect of KDM5B knockdown on the expression of KDM5B transcripts using qRT-PCR. **e** Loss of KDM5B function altered hsa-miR-448 mRNA expression in MDA-MB-231 cells. MDA-MB-231 cells were infected with scramble (231 V), shKDM5B clone II (231 II) or clone III (231 III) for 72 h. The transcript levels of KDM5B and hsa-miR-448 were assessed by qRT-PCR. **f** The mRNA expression of KDM5B and hsa-miR-448 is inversely correlated. Data are representative of 3 independent experiments and analyzed by student’s t-test. All data are shown as mean ± SEM. **p < 0.01, ***p < 0.001

Since the intent of our work was to proffer a clinically applicable solution and not just highlight a problem, we used a combination of small molecule inhibitors, genetic ablation or sncRNA to systematically screen for an effective therapeutic approach that targets KDM5B or MALAT1, and improve clinical outcome. Using bioinformatics approach, we also probed available freely accessible miR databases for miRs associated with KDM5B expression and came up with a panel of miRs (data not provided). From our literature-based functional analyses, deductive association, and miR - mRNA alignment, we narrowed down on hsa-miR-448 (Fig. 8). Our bioinformatics prediction was validated by using the sequence-based RNA-protein interaction algorithm described earlier in section (KDM5B interacts with MALAT1 to modulate its expression in triple negative breast carcinoma cells and consequently facilitate invasion and associated metastatic activities). We found that the 5′ end of hsa-miR-448 perfectly aligned with and bonded to the 3′ end of KDM5B (Fig. 8a & b), with a RF and SVM interaction classifier score of 70 and 81 % respectively (Fig. 8c). Notable is the finding that the hsa-miR-448 - KDM5B – MALAT1 complex formability ranged from 75 to 99.7 % based on RF and SVM classification (Fig. 8c). Our miRs screening showed that hsa-miR-448 was most downregulated among RT-qPCR-probed miRs in the KDM5B-overexpressing cells (data not provided). In addition, the ectopic expression of KDM5B and its downstream effector gene, MALAT1, inversely corrected with that of hsa-miR-448 in the triple negative breast cancer cells (Fig. 8d–f). These results were indicative of hsa-miR-448 critical role in the negative regulation of KDM5B expression and consequently, TNBC progression. Validative experiments showing that hsa-miR-448 improves clinical outcome and enhances chemosensitivity in breast carcinoma, in vivo, are already ongoing.

## Discussion

Triple negative breast cancer (TNBC) defines a group of hormone-deficient breast carcinoma associated with very aggressive tumor biology, dearth of documented targeted therapy and almost invariably, unfavourable clinical outcome [29]. Recently, the identification, validation and integration of novel biomarkers with current diagnostic or prognostic practices in TNBC clinics have been the subject of several studies, with the aim of proffering better patient stratification and more effective therapeutic strategy. In this study, we demonstrated the critical roles of KDM5B and its downstream target MALAT1 in triple negative breast cancer metastatic phenotype and clinical prognosis, based on the following observations: (i.) KDM5B was more abundant in the proteome of the highly metastatic TNBC cells, (ii.) KDM5B was over-expressed in highly invasive TNBC MDA-MB-231 and other metastatic cell lines, than in non-invasive MCF-7 and non-tumorigenic MCF-10A cells, (iii.) KDM5B ablation attenuated tumor cell migration, invasion, clonogenicity, and mammosphere formation ability of breast cancer cells, (iv.) KDM5B-silencing suppressed MALAT1 expression and inhibited cell proliferation, and (v.) human breast carcinoma samples exhibited the highest expression of KDM5B and MALAT1 in advanced stage (T3 and T4) cancer with associated poorer overall survival. These findings were concordant with our hypotheses that KDM5B through the modulation of MALAT1 expression is associated with breast tumorigenesis, progression and poor prognosis.

Recently, the role of epigenetic regulation in breast cancer biology, especially that of the histone lysine demethylases (KDMs), has been the subject of several studies [30]. This group of chromatin structure modifiers are increasingly shown to facilitate several steps of cancer progression. Several KDMs, including KDM5B have been implicated in tumor growth, angiogenesis, invasion, metastasis, and more recently, in tumor-related chemoresistance [31, 32]. KDM5B, a member of the JmjC/ARID domain-containing protein family, with restricted tissue distribution, specifically removes methyl residues from methylated lysine 4 of histone 3 (H3K4), consequently repressing gene transcription. This repression is through the discriminate binding of the AT-rich interactive domain (ARID) of KDM5B to CG-rich DNA sites, or its interaction with proteins at the DNA binding domain [33, 34]. In contrast to its physiologically low expression in normal adult tissue, except in the testes where it is highly expressed, aberrant expression of KDM5B has been demonstrated in skin, lung, prostate, bladder and recently in breast cancer tissues and cell lines, while KDM5B gene silencing, similar to knockdown of KDM3A, was shown to cause a significant G_1_/S transitional lag in MCF-7 breast tumor cells, suggesting its proliferative and tumorigenic activity [35–39]. Similar to KDM1A, KDM5B is involved in the silencing of breast tumor suppressors, including BRCA 1 [40], BRCA 2 [41], pRB [42], CAV1 [43], HOXA5 [44] and SFN [45], in addition to its active role in the signal transduction of hormone-regulated organs such as the ovaries, testes and breasts of gravid females, as well as, in transcriptional activation of androgen receptor (AR) [31, 33].

Recent studies have shown that the dissemination of malignant cells, as well as disease recurrence, is linked to the activity of a small subpopulation of cancer cells believed to possess tumor-growth initiating abilities [46, 47]. Based on the role of KDM5B in cancer stem cell-like events such as cell fate determination, self-renewal and enhanced cell motility [48, 49], we hypothesized that KDM5B is actively involved in the highly metastatic phenotype of TNBC cells.

Our immunohistochemistry analysis of metastatic human breast cancer samples indicated a positive correlation between KDM5B expression, advanced tumor stage and poor clinical outcome of patients (Figs. 1 and 2). This is suggestive of KDM5B’s ability to enhance tumor cell invasion, facilitate their homing and increase the clonogenicity of KDM5B-expressing cells. It is thus not unlikely that secondary site colonization by highly metastatic TNBC cells involves the active participation of KDM5B and its functional substrate, MALAT1. Our data revealed a correlative association between the expression and/or activity of KDM5B, MALAT1 and that of MALAT1-regulated effector genes, vimentin and snail (Figs. 3 and 5). In parallel experiments, KDM5B ablation downregulated MALAT1 expression and also blocked the MALAT1-induced expression of snail and vimentin (Fig. 6). Taken together, these data show that KDM5B via modulation of MALAT1 activity plays a critical role in the maintenance of TNBC invasive phenotype, and that the KDM5B–MALAT1 signalling axis regulates the activity of the metastatic EMT factors, such as snail and vimentin. These findings are consistent with previous findings in which MALAT1 was shown to positively modulated tumor cell motility by concomitantly regulating motility factors, including snail and vimentin [50, 51].

Furthermore, to validate the structural feasibility of KDM5B-MALAT1 complex formation, we used the GUI PyMOL software to demonstrate ligand docking coupled with limited binding site analysis (Fig. 7). In addition, we utilized the catRAPID graphic and strength RNA-protein interaction validation tool for confirmation of KDM5B-MALAT1 complex formation with KDM5B-MALAT1 interaction strength of 93 % (Fig. 7) based on interaction propensity rank of binding regions in the positive set (10201 interacting pairs). We propound that targeting these factors of breast tumor aggression, or their regulatory genes, might be a more effective therapeutic approach for combating metastasis and disease recurrence in breast cancer patients.

MicroRNAs (miRs) play very critical roles in the regulation of many eukaryotic genes and their associated bioprocesses, however, epigenetic dysregulation of these miR activity or expression has been implicated in several human carcinomas including breast carcinoma [52]. Differential expression of miRs in tumor versus non-tumor tissues, tumor tissue groups with varying degrees of invasiveness, or amongst tumor samples with favourable compared to poor clinical outcome, currently serve as vital template for the generation of miR signatures with potential diagnostic and/or prognostic value. Nevertheless, the biofunctional relevance of aberrant miR expression in triple negative breast carcinoma remained largely underexplored. Thus, we evaluated the interaction between KDM5B and the miR, hsa-miR-448.

Our results demonstrate that the expression of hsa-miR-448 is inversely correlated with that of KDM5B (Fig. 8). Mechanistically, miRs suppress the expression of their target genes by effecting proximity and facilitating interaction between RNAi-induced silencing complex (RISC) effector proteins and complementary sequences of the target mRNA [53]. In compliance with the consensual miRNA - mRNA rule of functional binding, nucleotide 2 to 7 of the 5′ region of hsa-miR-448 served as the interaction ‘seed’ region. The seed match of this evolutionarily conserved region of hsa-miR-448 bonding in a complementary manner with nucleotides in the 3′ untranslated region (3′UTR) of KDM5B was sufficient for KDM5B mRNA recognition and eventual degradation (Fig. 8). On the molecular level, we demonstrated that this process combines inhibition of translation, and mRNA degradation. Our data show that hsa-miR-448, with as little as 6 base pair (bp) match, significantly suppressed KDM5B expression (Fig. 8). Consistent with emerging model of miR-mediated gene silencing [54, 55], it is probable that this hsa-miR-448-induced suppression of KDM5B expression is sequel to the deadenylation and exonucleolytic cleavage of KDM5B mRNA.

We are not unaware of the limitations associated with the use of differential gene expression in the identification of miR targets, such as their link with several indirect alterations in transcript abundance, however, this pattern of gene expression utility in the identification of miR targets is in compliance with several seminal reports, including that which indicates that over 84 % of miR-mediated gene suppression was due to reduced mRNA levels [56–58].

## Conclusion

Unravelling the molecular mechanism of KDM5B expression in TNBC cells remains a work in progress, however, taken together, our findings indicate that KDM5B is an epigenetic modulator of MALAT1 activity and its downstream effector genes, SNAI and vimentin, as well as plays a critical role in tumor invasion, survival, and niche colonization by the highly metastatic TNBC cells. We demonstrated that KDM5B is a surrogate prognostic biomarker of breast cancer progression and represents a therapeutic target in metastatic breast cancer. Furthermore, we showed that the microRNA, hsa-miR-448 significantly inhibits MALAT-mediated oncogenic and metastatic potential by directly suppressing the expression of KDM5B in triple negative breast cancer, thus, alluding to the clinicopathophysiologic relevance of these findings. The dearth of an effective TNBC anticancer therapy necessitates the development of new therapeutic strategies such as miR replacement therapy. We posit that systemic hsa-miR-448 inoculation, targeting the expression and/or activities of KDM5B may be a stride in the right direction in combating the menace of triple negative breast cancer.

## Additional files

Additional file 1: Table S1. Quantification of GAPDH-normalized average gene expression in MCF10A OE, MDA-MB-231 WT and MDA-MB-231 KD cells with estimated fold change in expression. (DOCX 59 kb) Additional file 2: Figure S1. Relative expression of KDM5B, MALAT1, SNAIL, Vimentin and miR 448 normalized against GAPDH in MCF10A WT, MCF10A OE, MDA-MB-231 WT and MDA-MB-231 KD cells. Data are representative of 3 independent experiments and analyzed by student’s t-test. All data are shown as mean ± SEM. WT, wild type; OE, KDM5B overexpressed; KD, knockdown using shKDM5B clone II. (DOCX 519 kb) Additional file 3: Figure S2. KDM5B is preferentially expressed in TNBC tissues. (A) Box plot and (B) Heat map, showing expression of KDM5B and its associated genes in human breast invasive carcinoma. (C) KDM5B overexpression in TNBC is associated with poor prognosis. TCGA data obtained and analysed via UCSC genome browser. (DOCX 312 kb)
