# Supplementary material for: Aberrant KDM5B expression promotes aggressive breast cancer through MALAT1 overexpression and downregulation of hsa-miR-448
Source: BMC Cancer. 2016 Feb 25;16:160. doi: 10.1186/s12885-016-2108-5 (PMC4768424; doi:10.1186/s12885-016-2108-5)

Additional file 1: Table S1. Quantification of GAPDH-normalized average gene expression in MCF10A OE, MDA-MB-231 WT and MDA-MB-231 KD cells with estimated fold change in expression


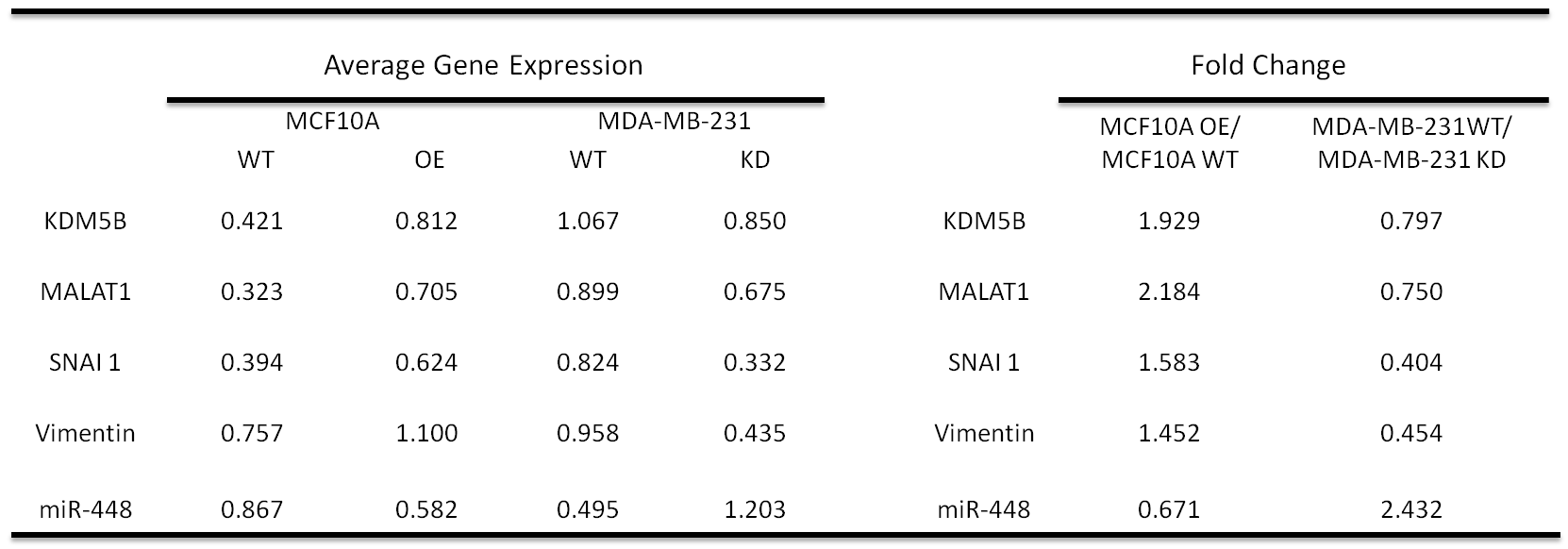

Supplement: Additional file 1: Table S1. — Quantification of GAPDH-normalized average gene expression in MCF10A OE, MDA-MB-231 WT and MDA-MB-231 KD cells with estimated fold change in expression. (DOCX 59 kb) [file 12885_2016_2108_MOESM1_ESM.docx]
