# Supplementary figures and images for: Aberrant KDM5B expression promotes aggressive breast cancer through MALAT1 overexpression and downregulation of hsa-miR-448
Source: BMC Cancer. 2016 Feb 25;16:160. doi: 10.1186/s12885-016-2108-5 (PMC4768424; doi:10.1186/s12885-016-2108-5)

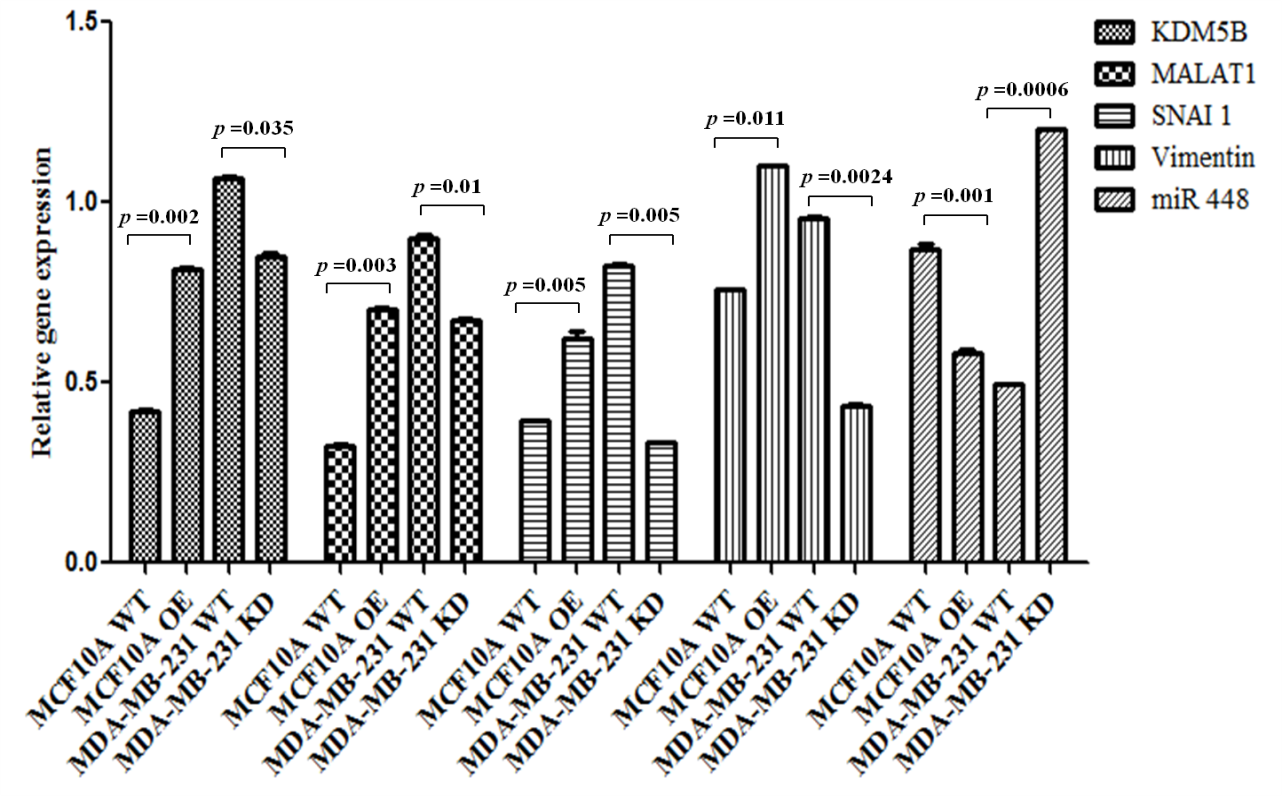


Additional file 2: Figure S1

Supplement: Additional file 2: Figure S1. — Relative expression of KDM5B, MALAT1, SNAIL, Vimentin and miR 448 normalized against GAPDH in MCF10A WT, MCF10A OE, MDA-MB-231 WT and MDA-MB-231 KD cells. Data are representative of 3 independent experiments and analyzed by student’s t-test. All data are shown as mean ± SEM. WT, wild type; OE, KDM5B overexpressed; KD, knockdown using shKDM5B clone II. (DOCX 519 kb) [file 12885_2016_2108_MOESM2_ESM.docx]

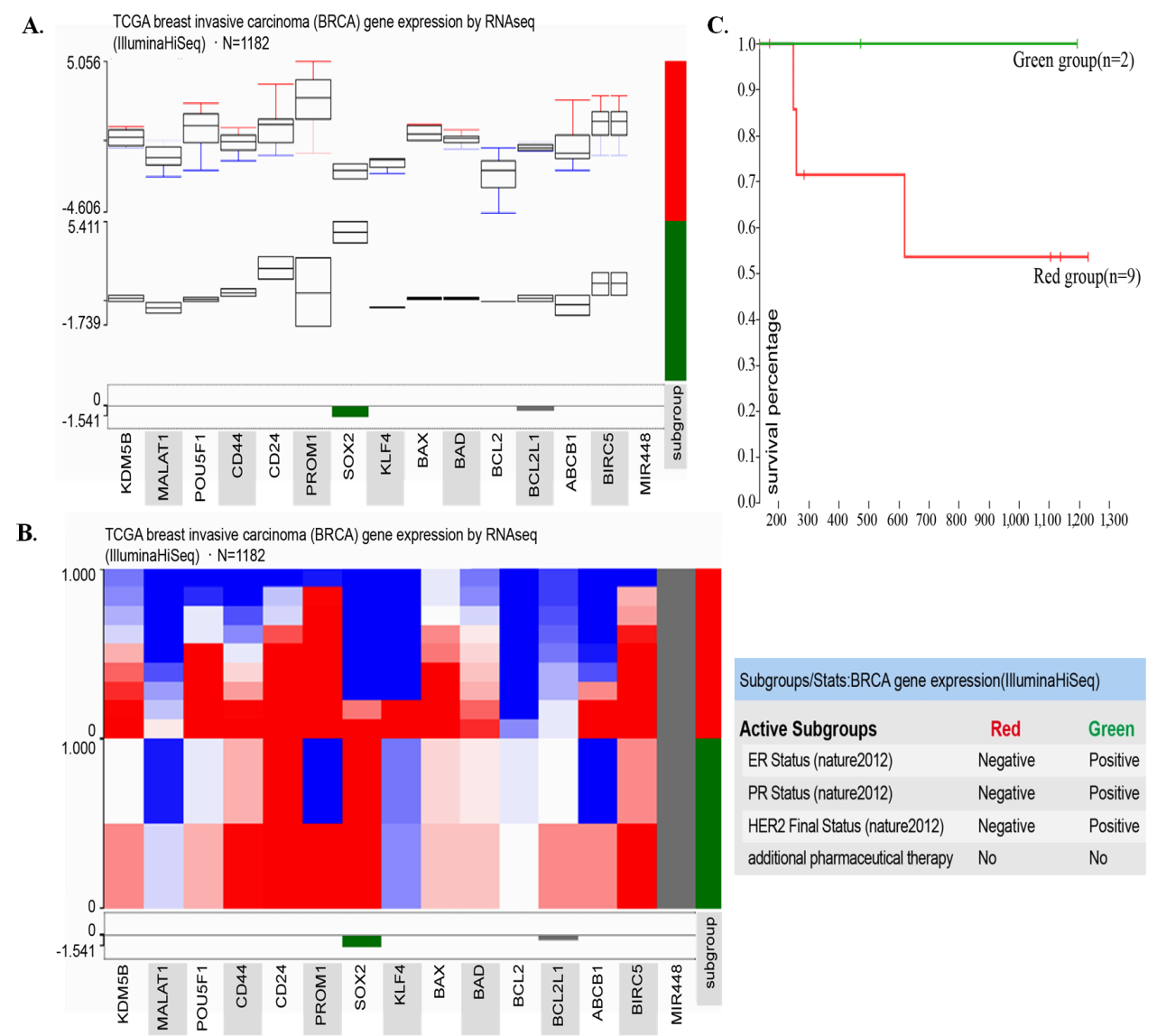


Additional file 3: Figure S2

Supplement: Additional file 3: Figure S2. — KDM5B is preferentially expressed in TNBC tissues. (A) Box plot and (B) Heat map, showing expression of KDM5B and its associated genes in human breast invasive carcinoma. (C) KDM5B overexpression in TNBC is associated with poor prognosis. TCGA data obtained and analysed via UCSC genome browser. (DOCX 312 kb) [file 12885_2016_2108_MOESM3_ESM.docx]
